# Physical Activity, Sedentary Behavior and Well-Being of Adults with Physical Disabilities and/or Chronic Diseases during the First Wave of the COVID-19 Pandemic: A Rapid Review

**DOI:** 10.3390/ijerph18126342

**Published:** 2021-06-11

**Authors:** Diederik R. de Boer, Femke Hoekstra, Kimberley I. M. Huetink, Trynke Hoekstra, Leonie A. Krops, Florentina J. Hettinga

**Affiliations:** 1 Centre for Human Movement Sciences, University Medical Center Groningen, University of Groningen, 9713 AV Groningen, The Netherlands; D.r.de.boer.1@student.rug.nl (D.R.d.B.); femke.hoekstra@ubc.ca (F.H.); k.i.m.huetink@student.rug.nl (K.I.M.H.); 2 Faculty of Health and Social Development, School of Health and Exercise Sciences, University of British Columbia Okanagan, Kelowna, BC V1V 1V7, Canada; 3 Department of Health Sciences and Amsterdam Public Health Research Institute, Vrije Universiteit Amsterdam, 1081 HV Amsterdam, The Netherlands; trynke.hoekstra@vu.nl; 4 Department of Rehabilitation Medicine, University Medical Center Groningen, University of Groningen, 9713 AV Groningen, The Netherlands; l.a.krops@umcg.nl; 5 Department of Sport, Exercise and Rehabilitation, Northumbria University, Newcastle NE1 8ST, UK

**Keywords:** coronavirus, rehabilitation, exercise, vulnerable populations, health

## Abstract

**Background:** People with physical disabilities and/or chronic diseases report lower levels of physical activity and well-being than the general population, which potentially is exacerbated through the COVID-19 pandemic. This study explored the international literature on physical activity, sedentary behavior and well-being in adults with physical disabilities and/or chronic diseases during the first wave of the pandemic. **Method:** In a rapid review, we included studies reporting on physical activity, sedentary behavior and/or well-being in adults with physical disabilities and/or chronic diseases. Four databases (Pubmed, CINAHL, PsycInfo, Embase) were searched for studies published until 30 September 2020. **Results:** We included twenty-nine studies involving eleven different types of disabilities or health conditions from twenty-one different countries. Twenty-six studies reported on physical activity, of which one reported an increase during the COVID-19 pandemic, four studies reported no difference, and twenty-one studies reported a decrease. Thirteen studies reported a decline in well-being. Only one study measured sedentary behavior, reporting an increase. **Conclusion:** Despite the variety in methods used, almost all studies reported negative impacts on physical activity and well-being in people with physical disabilities and/or chronic disease during the first wave of the pandemic. These findings highlight the importance of supporting this population, especially in times of crisis.

## 1. Introduction

In many developed countries, life expectancy has increased to over 80 years over the past century. At the same time, people have become increasingly physically inactive, leading to dramatic increases in lifestyle-related chronic diseases [1]. Recently, this was called the “*global pandemic of physical inactivity*” [2]. The financial burden of this physical inactivity pandemic was estimated to be USD 68 billion worldwide [3].

According to the updated World Health Organization (WHO) physical activity guidelines, all adults, including adults with disabilities or chronic diseases, should be active for 150–300 min at moderate intensity or 75–150 min at vigorous intensity aerobic per week to receive health benefits and reduce health risks [4]. Any bodily movement produced by skeletal muscles that requires energy expenditure could be classified under physical activity [4]. However, the majority of adults with disabilities do not meet these guidelines [5]. The WHO defined physical disabilities as “*an umbrella term for motor impairments, activity limitations and participation restrictions. It denotes the negative aspects of the interaction between an individual and that individual’s contextual factors*” [6]. In comparison with adults without disabilities or diseases, adults with physical disabilities or chronic diseases (such as cardiovascular or respiratory diseases) are, on average, less physically active and more sedentary [5,7]. To illustrate, it has been reported that 47% of the people with disabilities are inactive compared with 26% of the people without disabilities [5].

This is alarming, as physical activity, including sports, exercise, leisure time physical activity and active transport, is associated with many health benefits for people with physical disabilities and/or chronic diseases [8]. Being physically active decreases the risk for all-cause mortality and the development of several chronic diseases, such as coronary heart disease, hypertension, several types of cancer, type 2 diabetes mellitus, Alzheimer’s disease and dementia, in both adults without and with disabilities [5,9,10]. In people with physical disabilities, being physically active improves physical fitness, which results in maintenance of functional independence with ageing [11,12]. In people without disabilities, there is a strong association between sedentary behavior and the risk for mortality and developing chronic diseases, independent of physical activity level [13]. Sedentary behavior could be defined as “*any waking behavior characterized by an energy expenditure of 1.5 METs or lower while sitting, reclining or lying*” [4]. This specific association between sedentary behavior and health is not yet studied in people with physical disabilities. Furthermore, adequate physical activity positively affects self-reported well-being and mental health in people with physical disabilities, expressed as higher quality of life, lower anxiety and depression scores, a more positive body image and better self-appearance [14,15]. The WHO defined this well-being as “*the general term encompassing the total universe of human life domains, including physical, mental and social aspects, that make up what can be called a ‘good life’*” [6]. Another benefit of sufficient physical activity is that it has a positive effect on the immune system, by retaining metabolic balance, decreasing inflammation and increasing the number of lymphocytes [16,17]. These effects of physical activity on the immune system may be of particular importance in times of a virus pandemic.

After the first cases of the novel corona-type virus, named COVID-19 or Sars-CoV-2, were reported in December 2019, the WHO named COVID-19 as a pandemic on 11 March 2020 [18]. As a result of the pandemic, many countries subsequently went into (partial) lockdown, to the extent that for several countries, even outdoor activity was restricted for periods of time, and people were confined to their home environments for exercise [19]. Due to the lockdown, many sports facilities closed, which made it difficult to be physically active. By staying home, people avoided social contact. The outbreak of COVID-19 and the resulting lockdown have been generally associated with social and physical isolation [20,21,22,23] and have been found to impact on training and exercise in exercisers ranging from recreational to elite level [19]. It is precisely this kind of isolation that we have to beware of in people with physical disabilities and/or chronic diseases. In comparison with their peers without disabilities or diseases, adults with physical disabilities or chronic diseases experience generally higher levels of social isolation and loneliness and a lower level of perceived social support and social connectedness, and many already did so before the pandemic [24,25].

People with physical disabilities experience more and different barriers towards physical activity than their peers without disabilities [8]. People without physical disabilities experience personal barriers as the most important barrier leading them to not engage in sport or physical activity, such as lack of motivation or time. People with physical disabilities experience both personal (e.g., impaired mobility, fatigue and pain) as well as environmental barriers (e.g., lack of possibilities, lack of accessibility and transport) [8,26,27]. It is not unlikely that these barriers have expanded during the first wave of the COVID-19 pandemic. 

Therefore, we conducted a rapid review exploring physical activity, sedentary behavior and well-being during the first wave of the COVID-19 pandemic in people with physical disabilities and/or chronic diseases. The primary research question was:

1. What is reported in the international literature on physical activity behavior in adults with physical disabilities and/or chronic diseases during the first wave of COVID-19 pandemic?
  Secondary research questions were:
2. What is reported in the international literature on sedentary behavior in adults with physical disabilities and/or chronic diseases during the first wave of the COVID-19 pandemic?
3. What is reported in the international literature on well-being in people with physical disabilities and/or chronic diseases during the first wave of the COVID-19 pandemic?

## 2. Methods

### 2.1. Study Design

We conducted a rapid review because of the immediate relevance and need in the ongoing COVID-19 pandemic. A rapid review can be defined as “*a form of knowledge synthesis that accelerates the process of conducting a traditional systematic review through streamlining or omitting a variety of methods to produce evidence in a resource-efficient manner*” [28]. Our study methods and results were guided by and reported using the Preferred Reporting Items for Systematic reviews and Meta-Analyses (PRISMA) guidelines [29]. 

### 2.2. Search Strategy and Eligibility Criteria 

Four health databases (Pubmed, CINAHL, PsycINFO, Embase) were searched for relevant studies published between 1 December 2019 and 30 September 2020. The search strategy included the following keywords: (1) terms related to COVID-19: “*COVID-19*” OR “*Sars-CoV-2*” OR “*Coronavirus*” OR “*Corona virus*” and (2) terms related to physical activity, sedentary behavior and well-being: “*Physical activity*” OR “*Sport*” OR “*Sports*” OR “*Exercise*” OR “*Exercising*” OR “*Physical training*” OR “*Physical performance*” OR “*Sedentary behavior*” OR “*Sitting activity*” OR “*Sedentary inactivity*” OR “*Well-being*” OR “*Wellness*” OR “*Wealth*” OR “*Welfare*”. To capture a broad range of potentially relevant literature, we did not include terms related to the population group in our search strategy. Functions in the databases were used to search only in the titles and abstracts and to include only studies written in English. Table A1 in Appendix A outlines additional details of the search strategies for each database. 

We included primary studies that reported on the impact of the COVID-19 pandemic on physical activity, sedentary behavior and/or well-being in adults with a physical disability and/or chronic diseases. To be included in this review, the study had to report primary data on changes in physical activity, sedentary behavior and/or well-being in adults with a physical disability and/or chronic disease. Studies about people without a disability or chronic disease or children/youth were excluded. We also excluded studies about people with a visual, intellectual, aural or psychological disability. 

Table 1 presents further details on inclusion and exclusion criteria. 

### 2.3. Data Screening

The reference manager EndNote (EndNote X9 3.3, Clarivate Analytics, 160 Blackfriars Road, London, UK) and Excel were used to export and manage the results. The guidelines of Bramer et al. [30] were followed to remove duplicates. Title/abstract and full text screening were conducted by the first author (DB). Any uncertainties about eligibility criteria were discussed during a meeting with other team members (KH, FH, FJH), and final decisions about inclusion/exclusion were made accordingly. 

### 2.4. Data Extraction and Analyses

Data extraction was done by two team members (DB or KH) using Excel. The following data were extracted from the included studies: authors, year of publication, study design, study population, participant information (disability/ health condition, age, gender), physical activity/sedentary behavior/well-being construct, measures (e.g., questionnaire or accelerometer), key results related to the impact on physical activity/sedentary behavior/well-being during the pandemic and direction of the impact (positive, negative, no change). Aligning with our research questions, the findings on physical activity were presented separately from the findings on sedentary behavior and well-being. 

## 3. Results

**Figure 1:**
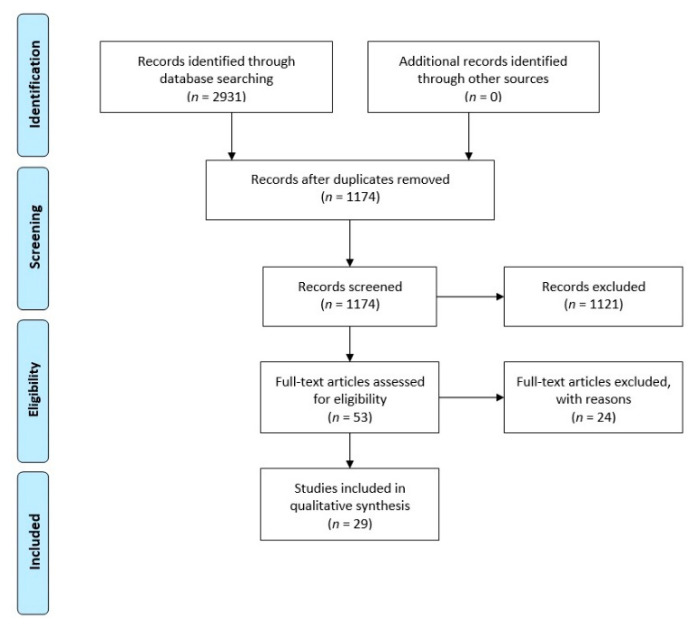
Flowchart of literature search.

The search strategy resulted in a total of 2931 articles identified from the four databases. After de-duplication, a total of 1174 unique articles remained. After screening of title and abstract, a total of 53 studies remained. From these 53 studies, 29 studies were included in this review after a full-text screening. Table A2 includes a list of excluded articles during full-text screening. Figure 1 presents a flowchart of the search procedure. 

The study characteristics are summarized and presented in Table 2. Twenty-one of the twenty-nine studies (72%) were cross-sectional studies (CS) [31,32,33,34,35,36,37,38,39,40,41,42,43,44,45,46,47,48,49,50,51], four studies (14%) were observational studies (OS) [52,53,54,55], and four studies (14%) were prospective cohort studies (PC) [56,57,58,59], whereas three were a prospective cohort study within an ongoing randomized clinical trial (PC-RCT) [57,58,59]. The studies were conducted in twenty-one different countries across four continents. Six studies (21%) were conducted in Italy [35,45,52,54,55,58], three (10%) in each of India [39,43,53] and the USA [33,48,58], two (6,9%) in each of Belgium [38,58], China [37,51], France [34,57] and the Netherlands [49,50] and one (3%) was conducted in each of Austria [56], Brazil [32], Canada [58], Czech Republic [59], Denmark [58], Egypt [46], Israel [36], Japan [40], Kuwait [31], Pakistan [44], South Korea [47], Spain [42], Switzerland [41], the UK [58] and one worldwide [33]. We included studies focusing on the following types of disabilities or health conditions: diabetes mellitus (*n* = 8; 28% [32,39,40,42,43,51,52,53]), Parkinson’s disease (*n* = 5; 17% [33,45,46,47,50]), cardiovascular diseases (*n* = 5; 17% [34,54,55,57,59]), multiple chronic diseases (*n* = 3; 10% [36,44,48]), cystic fibrosis (*n* = 2; 7% [38,41]), osteoarthritis (*n* = 1; 3.4% [56]), multiple sclerosis (*n* = 1; 3.4% [58]), neuromuscular diseases (*n* = 1; 3% [35]), hereditary spastic paraplegia (*n* = 1; 3% [49]), skin diseases (*n* = 1; 3% [37]) and migraine (*n* = 1; 3% [31]). The number of participants ranged from 24 [55] to 9016 [51]. 

### 3.1. Physical Activity (Primary Research Question) 

Twenty-six studies (81%) reported findings about physical activity during the first wave of the COVID-19 pandemic [31,32,33,34,35,36,38,39,40,41,42,43,44,45,46,47,49,50,51,52,54,55,56,57,59]. These studies included 23,710 individuals with nine different types of disabilities or chronic diseases. One study (4%; 1 out of 26) including adults with diabetes [51] reported an increase in physical activity during the COVID-19 pandemic. Twenty-five studies (96%; 25 out of 26) reported no difference or a decrease in physical activity. The key findings regarding physical activity behavior during the pandemic are summarized in Table 3. A variety of physical activity constructs (e.g., daily physical activity, number of steps, moderate-intensity and vigorous-intensity activities) was used to assess physical activity. Twenty-three studies used self-reported measures and four used accelerometer-based measures. Across all included studies, constructs of physical activity were measured with thirteen different measures (see Table 4 for an overview, see Table S1 for a more detailed overview).

### 3.2. Sedentary Behavior and Well-Being (Secondary Research Questions) 

Only one study [42] reported on changes in sedentary behavior during the first wave of the pandemic (see Table 3). This study reported that adults with type 2 Diabetes Mellitus in Spain increased sitting time during the COVID-19 pandemic compared with before the pandemic. 

Thirteen of the included studies (45%) reported findings on changes in well-being during the pandemic [36,37,38,43,44,45,46,48,49,50,57,58]. These studies included 2466 individuals with nine different types of disabilities or health conditions. All thirteen studies reported a negative change in one or more constructs related to well-being of adults with physical disabilities or chronic diseases during the first wave of the COVID-19 pandemic. These findings are summarized in Table 5. Across the thirteen studies, nine different well-being constructs (anxiety, depression, loneliness, mental health, overall health, pain, quality of life, stress, well-being) were reported. Table 6 provides an overview of the well-being constructs.

## 4. Discussion

This rapid review provides an overview of studies reporting on physical activity, sedentary behavior and well-being in people with physical disabilities and/or chronic diseases during the first wave of the COVID-19 pandemic. In the short time after the COVID-19 outbreak, we identified already twenty-nine studies including different types of physical disabilities and chronic diseases from twenty-two different countries on four different continents. Despite the large variation in study contexts and methodologies, almost all studies reported a negative impact on physical activity, sedentary behavior and well-being during the first wave of the COVID-19 pandemic. 

### 4.1. Impact on Physical Activity during the COVID-19 Pandemic 

Twenty-six studies reported on physical activity during the first wave of the pandemic. Almost all studies demonstrated a negative impact on the level of physical activity. This negative impact on physical activity is in accordance with a systematic review summarizing sixty-four articles on physical activity change during the first wave of COVID-19 in the general population [60]. An earlier rapid review, studying the broader impact of COVID-19 on health and participation also found a decrease of physical activity in people with neuromuscular disease and chronic pain [23]. This negative impact on physical activity can probably be explained by the many barriers regarding physical activity that people with (or without) physical disabilities may face [8]. Many of these influencing factors, such as social support, professional assistance, and availability of equipment and transportation, became less available in many countries due to lockdown restrictions, including the closing of sports facilities. It is important to note that this is a worldwide review and that lockdown restrictions varied between countries. People in some countries were obliged to stay home, while people in other countries were still able to be active outside, a finding that also came forward in the study by Washif et al. (under review) [19]. Although not studied, it is likely that the magnitude of impact of COVID-19 restrictions on physical activity, sedentary behavior and wellbeing, summarized in this rapid review, may be associated with the severity of lockdown restrictions.

Included studies in this review used a variety of methodologies (Table 3) and physical activity measures (Table 4). The majority of the studies assessed the self-reported difference in the degree of physical activity between the situation before the pandemic compared with the situation during lockdown. Many questionnaires were investigator-developed and/or non-validated. However, the almost unanimous negative impact on physical activity during the pandemic found in this review, shows again the importance of more attention and guidance for people with physical disabilities and/or chronic diseases because it is precisely this group that can benefit a great deal from regular physical activity [5,7,61].

### 4.2. Impact on Sedentary Behavior during the COVID-19 Pandemic 

The secondary outcome of this rapid review related to the impact of sedentary behavior during the COVID-19 pandemic. Surprisingly, sedentary behavior was measured in only one of the included studies. This one study [42] reported a negative impact of the COVID-19 pandemic on sedentary behavior [60]. A similar trend has been reported in the general population. In the same publication period, only two articles have been identified reporting on the impact of sedentary behavior in people with medical conditions [62,63]. It is worrying that sedentary behavior was studied so little during the pandemic. Work-from-home policies that were implemented in many countries were likely to increase screen time and thus may have encouraged people to adopt sedentary behavior. Sedentary behavior is known to be a health risk independent of physical activity and therefore it is advised be studied as a separate behavior. The study by Stockwell et al. reported that the majority of the studies that measured sedentary behavior in people with medical conditions used non-validated questionnaires as well [60]. This might indicate that, in comparison with physical activity, it remains difficult to adequately measure sedentary behavior, especially among special populations such as people with disabilities and/or chronic diseases. Therefore, more research on (how to measure) sedentary behavior in specific populations is needed to better understand how to protect this population group against the risks of sedentary behavior, both during and after pandemics the magnitude of COVID-19.

### 4.3. Impact on Well-Being during the COVID-19 Pandemic

All of the identified studies in this review reporting on well-being demonstrated a negative impact on one or more constructs related to well-being during the first wave of the COVID-19 pandemic. Our findings align with other recent reviews reporting the negative impact of a variety of well-being constructs during the COVID-19 pandemic among different populations [20,21,22,23]. Interestingly, a recent review found that regular physical activity was related to lower levels of depression and anxiety in the general population during the COVID-19 pandemic [22] but that the pandemic had increased levels of depression and anxiety. Based on literature before the COVID-19 pandemic [14,15], it can be expected that regular physical activity may also be associated with positive outcomes on a variety of well-being constructs during the pandemic. This highlights again the importance of promoting physical activity in people with physical disabilities and/or chronic diseases. 

Included studies in this review reported on a variety of well-being constructs using a variety of measurement tools, which is not surprising given the multidimensional character of the well-being. While there is a lack of consensus in the literature on how to define and operationalize well-being, which might partly depend on the research field and/or focus of a study [64], we used a general definition capturing both mental and physical components of well-being. This might be a contributing factor to the variety of measurement tools that was found [6]. Additionally, before COVID-19, measuring well-being was already more difficult for people with a disability compared with their peers without a disability [24,25]. The variety of measurement instruments used in the studies we included in this review made it difficult to compare their effect sizes directly. Moreover, our results clearly illustrate a negative impact on well-being of people with a physical disability and/or chronic disease during the first wave of the COVID-19 pandemic, regardless of how well-being is operationalized. This finding shows the importance of guidance and mental support, especially in times of crisis.

### 4.4. Scientific and Practical Implications

We were able to identify 29 studies conducted in 21 different countries and among 11 different groups of diagnosis. Another review studying changes in physical activity and sedentary behavior from before to during the pandemic lockdown amongst healthy children, adolescents and adults was able to include 66 studies [60]. Both showed decreased physical activity levels in almost all included studies, most likely indicating additional barriers for engagement in an active lifestyle. This is particularly relevant for populations with disabilities and chronic diseases who already experience substantial barriers to physical activity engagement [8,26,27]. Our rapid review is, to the best of our knowledge, the first study that has investigated and summarized physical activity, sedentary behavior and well-being in people with physical disabilities and/or chronic diseases during the first wave of the COVID-19 pandemic, establishing the need for an additional focus on vulnerable populations and physical activity stimulation. Digital technology and home-based alternatives have been mentioned as ways to provide potential support mechanisms to recreational athletes during a pandemic [18]. This could be promising to include in tailored programs to promote physical activity in persons with disabilities and/or chronic disease as well, though tailoring to their specific barriers will be needed. The results of this study show practical implications for medical support staff and policy makers. Policy makers might want to give special attention to this group, especially in times of crises. 

### 4.5. Limitations

Some limitations need to be addressed. The first limitation concerns our search strategy. While our strategy included various terms to capture “physical activity” and “sedentary behavior” constructs, only a few terms were included to capture articles reporting on “well-being”. As such, we may have missed relevant articles reporting on the impact of well-being during the pandemic, possibly impacting the rigor of this review. When specifically interested in well-being, we recommend using a more comprehensive search strategy including a variety of terms to capture the well-being construct. The second limitation concerns the quality of the studies. Many of the included studies were cross-sectional studies across different setting using a variety of measurement instruments that were not validated for the population concerned, indicating that findings should be interpreted with caution. On the other hand, the fact that we were able to include already 29 studies, may highlight the urgency of studying the physical activity and well-being of people with physical disabilities and/or chronic diseases during, but perhaps also after, the pandemic. Despite these limitations, the directions of the findings (i.e., negative impact on physical activity, sedentary behavior and well-being) were consistent across almost all of the included studies. Lastly, this review focused on the impacts during the first wave of the pandemic. It is possible that there are or were other behaviors affected in subsequent waves of the COVID-19 pandemic.

## 5. Conclusions

Despite the large variation in methods of measuring physical activity and well-being, the vast majority of the included studies reported a negative impact on physical activity and well-being in adults with physical disabilities and/or chronic diseases during the first wave of the COVID-19 pandemic. Unfortunately, the impact on sedentary behavior was barely measured. The consistent findings of the negative impact during the COVID-19 pandemic that are reported in this rapid review illustrate the need to provide (additional) support and guidance to people with a physical disability and/or chronic disease to help them become and stay physically active and well during a pandemic. 

## Figures and Tables

**Table 1:** Inclusion and exclusion criteria.

	Inclusion Criteria	Exclusion Criteria
**General**	- The study reports on the impact of the COVID-19 pandemic on physical activity, sedentary behavior, and/or well-being in adults (>18 years) with a physical disability and/or chronic diseases.	
**Population**	- The study is about people with a physical disability and/or chronic disease. Physical disability is defined here as “an umbrella term for motor impairments, activity limitations and participation restrictions. It denotes the negative aspects of the interaction between an individual and that individual’s contextual factors” [6].	- The study is about people without a disability and/or chronic disease. - The study is about people with a visual, hearing, intellectual and/or psychological disability. - The study is about children and/or youth.
**Intervention**	- Not applicable	
**Comparison**	- The study compares the situation before the COVID-19 pandemic with the situation in the COVID-19 pandemic.	
**Outcomes**	- The study reports on the impact of COVID-19 pandemic on physical activity, sedentary behavior AND/OR well-being. - Physical activity is defined as “any bodily movement produced by skeletal muscles that requires energy expenditure” [4]. - Sedentary behavior is defined as “any waking behavior characterized by an energy expenditure of 1.5 METs or lower while sitting, reclining or lying” [4]. - Well-being is defined as “the general term encompassing the total universe of human life domains, including physical, mental and social aspects, that make up what can be called a ‘good life’” [6].	
**Study design**	- The study is a primary data study (e.g., cross-sectional, randomized controlled trials, observational etc.).	- The study is an integrative method (e.g., reviews, meta-analysis, editorials, commentary etc.).
**Other**	- The study is published between 1 December 2019 and 30 September 2020. - The study is published in English.	- The study is published after 30 September 2020.

**Table 2:** The study characteristics of the included studies.

Author and Year	Country	Design	Type of Disability or Health Condition	Participants (n)	Age (Year) and Gender
Barone et al. (2020) [32]	Brazil	CS	Diabetes Mellitus	1701	Age: 18–30: 395, 30–40: 453, 40–50: 351, 50–60: 271, 60–70: 164, 70–80: 59, 80>: 8 Gender: M = 414, F = 1285
Khader et al. (2020) [39]	India	CS	Diabetes Mellitus	1510	Age: 41.6 Gender: M = 963, F = 543
Yan et al. (2020) [51]	China	CS	Diabetes Mellitus	9016 (DM: 585, no DM: 8431)	Age: 18–80 Gender: M = 3839, F = 5177
Assaloni et al. (2020) [52]	Italy	OS	Type 1 Diabetes Mellitus	154	Age: 44.8 ± 12.5 Gender: M = 84, F = 70
Khare et al. (2020) [53]	India	OS	Type 2 Diabetes Mellitus	143	Age: 54.68 ± 9.22 Gender: M = 91, F = 52
Munekawa et al. (2020) [40]	Japan	CS	Type 2 Diabetes Mellitus	203	Age: 67.4 ± 11.3 Gender: M = 126, F = 77
Ruiz-Roso et al. (2020) [42]	Spain	CS	Type 2 Diabetes Mellitus	72	Age: 63 (44–77) Gender: M = 35, F = 37
Sankar et al. (2020) [43]	India	CS	Type 2 Diabetes Mellitus	110	Age: 58.7 ± 10.8 Gender: M = 42, F = 68
Brown et al. (2020) [33]	USA/World	CS	Parkinson’s disease (PD)	7209 (PD: 5429, No PD: 1780)	Age: 19–95 Gender: M = 3445, F = 3764
Schirinzi et al. (2020) [45]	Italy	CS	Parkinson’s disease	74	Age: 61.3 ± 9.3 Gender: M = 37, F = 37
Shalash et al. (2020) [46]	Egypt	CS	Parkinson’s disease	58 (PD: 38, No PD: 20)	Age: PD: 55.579 ± 9.956, No PD: 55.550 ± 5.708 Gender: M = 43, F = 15
Song et al. (2020) [47]	South Korea	CS	Parkinson’s disease	100	Age: 70 (62.3–76.0) Gender: M = 54, F = 46
Van der Heide et al. (2020) [50]	The Netherlands	CS	Parkinson’s disease	358	Age: 62.8 ± 9.0 Gender: M = 220, F = 138
Chagué et al. (2020) [57]	France	PC-RCT	Congestive heart failure	124	Age: 71.0 ± 4.0 Gender: M = 75, F = 49
Vetrovsky et al. (2020) [59]	Czech Republic	PC-RCT	Heart failure	26	Age: 58.8 ± 9.8 Gender: M = 18, F = 8
Malanchini et al. (2020) [54]	Italy	OS	Chronic cardiovascular disease	184	Age: 67 ± 14 Gender: M = 134, F = 50
Sassone et al. (2020) [55]	Italy	OS	Implantable cardioverter-defibrillators	24	Age: 72 ± 10 Gender: M = 17, F = 7
Cransac-Miet et al. (2020) [34]	France	CS	Chronic Coronary Syndromes	195	Age: 65.5 ± 11.1 Gender: M = 119, F = 76
Elran-Barak et al. (2020) [36]	Israel	CS	Chronic diseases	315	Age: 18–45: 60, 46–55: 43, 56–65: 69, 66–75: 107, 76>: 33 Gender: M = 121, F = 178
Saqib et al. (2020) [44]	Pakistan	CS	Chronic diseases	181	Age: 18–35: 75, 36–55: 52, 55>: 54 Gender: M = 109, F = 72
Umucu et al. (2020) [48]	USA	CS	Self-reported disabilities and chronic conditions	269	Age: 39.37 ± 12.18 Gender: M = 151, F = 118
Havermans et al. (2020) [38]	Belgium	CS	Cystic Fibrosis	219	Age: 16–67 Gender: M = 86, F = 133
Radtke et al. (2020) [41]	Switzerland	CS	Cystic Fibrosis	327	Age: 72.5% <40, 27.5% >40 Gender: M = 171, F = 155
Chiaravalloti et al. (2020) [58]	Italy/UK/Canada/Denmark/Belgium/USA	PC-RCT	Progressive Multiple Sclerosis	131	Age: 52,1 ± 9.6 Gender: M = 48, F = 83
Endstrasser et al. (2020) [56]	Austria	PC	Osteoarthritis	63	Age: 62.4 ± 11.84 Gender: M = 35, F = 28
Di Stefano et al. (2020) [35]	Italy	CS	Neuromuscular diseases	268 (NM: 149, No NM: 119)	Age: 57.3 ± 13.7 (NM) 56 ± 6.8 (no NM) Gender: M = 176, F = 92
Van de Venis et al. (2020) [49]	The Netherlands	CS	Hereditary spastic paraplegia	58	Age: 57 (range 30–77) Gender: M = 27, F = 31
Guo et al. (2020) [37]	China	CS	Skin diseases	506	Age: 33.5 ± 14.0 Gender: M = 217, F = 289
Al-Hashel et al. (2020) [31]	Kuwait	CS	Migraine	1018	Age: <20: 38, 20–40: 733, 40–60: 235, 60>: 12 Gender: M = 160, F = 858

Note: cross-sectional study, CS; observational study, OS; prospective cohort study within an ongoing randomized clinical trial, PC-RCT; prospective cohort study, PC; male, M; female, F.

**Table 3:** Key findings regarding physical activity during the first wave of the COVID-19 pandemic.

Author and Year	Type of Disability or Health Condition	PA Construct	Method	Primary Results	Change in PA *
Barone et al. (2020) [32]	Diabetes Mellitus	Change in PA	5-Likert scale question	59.5% reported a decrease in PA.	−
Khader et al. (2020) [39]	Diabetes Mellitus	Change in PA	3-Likert scale question	69.07% reported a decrease in PA.	−
Yan et al. (2020) [51]	Diabetes Mellitus	Changes in PA	International Physical Activity Questionnaire (IPAQ)	67.7% with diabetes (vs. 41.2% without diabetes) reported an increased level of PA.	+
Assaloni et al. (2020) [52]	Type 1 Diabetes Mellitus	Type of exercise Godin Scale Score Minutes of exercise Steps number	Godin-Leisure Time Exercise questionnaire (GLTEQ), Activity Tracker	Significant decrease in perceived and measured PA level.	−
khare et al. (2020) [53]	Type 2 Diabetes Mellitus	Change in type Change in timing Change in duration	2-Likert scale question	80.42% reported a change in type. 72.72% reported a change in timing. 60.84% reported a change in duration.	−
Munekawa et al. (2020) [40]	Type 2 Diabetes Mellitus	Change in exercise	Visual analogue scale (VAS)	53.69% reported a decrease in exercise level. Mean score of 3.7 (0: considerably reduced to 10: considerably increased)	−
Ruiz-Roso et al. (2020) [42]	Type 2 Diabetes Mellitus	Change in PA	IPAQ	Significant increase in the daily hours that the participants of the study were sitting without doing any PA at all. Significant decrease of the average minutes per week spent walking. Decline in the average weekly time spent doing any type of moderate physical activity.	−
Sankar et al. (2020) [43]	Type 2 Diabetes Mellitus	Change in PA	Face-to-face interview	82.7% reported no major change in PA.	
Brown et al. (2020) [33]	Parkinson’s disease	Change in exercise	4-Likert scale question	21% reported a cancelled/disrupted exercise. 7.9% reported a postponed exercise. 41% reported an alternative conducted exercise. 30% not reported any changes in exercise.	−/*
Schirinzi et al. (2020) [45]	Parkinson’s disease	Motor activity habits	International Physical Activity Questionnaire—Short Form (IPAQ-SF)	No change in total patients playing sports.	*
Shalash et al. (2020) [46]	Parkinson’s disease	Change in PA	IPAQ-SF, Parkinson’s Disease questionnaire (PDQ39), 2-Likert scale COVID questions	Significant decline in physical activity. Compared with control group: significant worse moderate physical activity, walking and total IPAQ. 68.4% of the patients reported decline of PA.	−
Song et al. (2020) [47]	Parkinson’s disease	Change in exercise (amount, duration and frequency)	Physical Activity Scale of the Elderly (PASE) questionnaire	Significant decrease in the amount of exercise.	−
Van der Heide et al. (2020) [50]	Parkinson’s disease	Change in PA	5-Likert scale question	46.6% were physically less active.	−
Chagué et al. (2020) [57]	Congestive heart failure	Change in PA	Telephone interview	41.9% reported a decreased PA.	−
Vetrovsky et al. (2020) [59]	Heart failure	Daily number of steps	Wrist-worn accelerometer	16% decrease of daily step count.	−
Malanchini et al. (2020) [54]	Chronic cardiovascular disease	Activity level (h/day)	Implanted devices	Decrease in PA of 0.5 h per day, a decrease of more than 25% compared with the activity during the pre-lockdown period and reference period.	−
Sassone et al. (2020) [55]	Implantable cardioverter-defibrillators	Change in PA	Implantable cardioverter-defibrillator	Mean 25% reduction of PA was observed.	−
Cransac-Miet et al. (2020) [34]	Chronic Coronary Syndromes	Change in PA	Telephone interview	45% declared >25% reduction in PA.	−
Elran-Barak et al. (2020) [36]	Chronic diseases	Level of PA	Adapted Medical Outcomes Study-Short Form 36 items (SF-36 MOS)	Significant decrease in PA.	−
Saqib et al. (2020) [44]	Chronic diseases	Change in daily exercise	2-Likert scale question	66% could not continue their daily exercise.	−
Havermans et al. (2020) [38]	Cystic Fibrosis	Change in exercise	2-Likert scale (yes/no)	53.2% of the adult CF patients reported they were not exercising more.	−/*
Radtke et al. (2020) [41]	Cystic Fibrosis	Change in PA	VAS	44.8% reported decreased PA.	−
Endstrasser et al. (2020) [56]	Osteoarthritis	Change in daily activity	Tegner activity scale (TAS)	Significant decreased level of activity.	−
Di Stefano et al. (2020) [35]	Neuromuscular diseases	Total PA level MVPA level (moderate-intensity and vigorous-intensity)	IPAQ-SF (adapted version)	Significant reduction of PA was reported for walking activity, total PA level and MVPA level, while no difference was found for vigorous-intensity PA and moderate-intensity PA.	−
Van de Venis et al. (2020) [49]	Hereditary spastic paraplegia	Change in PA	5-Likert scale question	74% reported a reduction of PA.	−
Al-Hashel et al. (2020) [31]	Migraine	Level of exercise	2-Likert scale question	79.7% reported an increased lack of regular exercise.	−

Note: * Change in physical activity during the COVID-19 pandemic compared with before the pandemic. A positive change (+) indicates an increase in physical activity, no change (*) indicates no change in physical activity and a negative change (−) indicates a decrease in physical activity during the COVID-19 pandemic compared with before the start of the pandemic.

**Table 4:** Different physical activity measurements used in the included studies.

	Self-Reported Measurements										Accelerometry			
Author and Year	LS	GLTEQ	IPAQ	IPAQ-SF	IV	PD Q39	PASE	SF-36 MOS	TAS	VAS	AT	ID	AM	Change in PA *
Barone et al. (2020) [32]	✓													−
Khader et al. (2020) [39]	✓													−
Yan et al. (2020) [51]			✓											+
Assaloni et al. (2020) [52]		✓									✓			−
Khare et al. (2020) [53]	✓													−
Munekawa et al. (2020) [40]										✓				−
Ruiz-Roso et al. (2020) [42]			✓											−
Sankar et al. (2020) [43]					✓									*
Brown et al. (2020) [33]	✓													−/*
Schirinzi et al. (2020) [45]				✓										*
Shalash et al. (2020) [46]	✓			✓		✓								−
Song et al. (2020) [47]							✓							−
Van der Heide et al. (2020) [50]	✓													−
Chagué et al. (2020) [57]					✓									−
Vetrovs-ky et al. (2020) [59]													✓	−
Malanchini et al. (2020) [54]												✓		−
Sassone et al. (2020) [55]												✓		−
Cransac-Miet et al. (2020) [34]					✓									−
Elran-Barak et al. (2020) [36]								✓						−
Saqib et al. (2020) [44]	✓													−
Havermans et al. (2020) [38]	✓													−/*
Radtke et al. (2020) [41]										✓				−
Endstrasser et al. (2020) [56]									✓					−
Di Stefa-no et al. (2020) [35]				✓										−
Van de Venis et al. (2020) [49]	✓													−
Al-Hashel et al. (2020) [31]	✓													−

Note: * Change in physical activity during the COVID-19 pandemic compared with before the pandemic. A positive change (+) indicates an increase in physical activity, no change (*) indicates no change in physical activity and a negative change (−) indicates a decrease in physical activity during the COVID-19 pandemic compared with before the start of the pandemic. Likert scale, LS; Godin-Leisure Time Exercise questionnaire, GLTEQ; International Physical Activity Questionnaire, IPAQ; International Physical Activity Questionnaire—Short Form, IPAQ-SF; Interview, IV; Parkinson’s Disease questionnaire, PDQ39; Physical Activity Scale of the Elderly, PASE; Medical Outcomes Study-Short Form 36 items, SF-36 MOS; Tegner activity scale, TAS; Visual analogue scale, VAS; activity tracker, AT; implanted devices, ID; accelerometer, AM.

**Table 5:** Key findings regarding well-being during the first wave of the COVID-19 pandemic.

Author and Year	Type of Disability or Health Condition	WB Constructs	Method	Primary Results	Change in Well-Being *
Sankar et al. (2020) [43]	Type 2 Diabetes Mellitus	Stress Anxiety	Hospital Anxiety and Depression Scale (HADS)	15.5% increased mental stress and higher anxiety levels.	−
Schirinzi et al. (2020) [45]	Parkinson’s disease	Depression	Parkinson’s Well-Being Map (PWBM), Beck Depression Index (BDI)	59.5% perception of worsening in global health during COVID. Worsening patients have a significant higher PWBM and BDI score.	−
Shalash et al. (2020) [46]	Parkinson’s disease	Mental health Health care	Depression, Anxiety, and Stress scale-21 (DASS-21), PD questionnaire (PDQ39), 2-Likert scale COVID questions	Compared with control group: significant worse stress, depression, anxiety and total DASS. 52.6% reported anxiety/stress due to COVID-19.	−
Van der Heide et al. (2020) [50]	Parkinson’s disease	Perceived stress PD symptom severity Stressor load	DynaCORE-C, Perceived Stress Scale (PSS), Unified Parkinson’s Disease Rating Scale part Ib and II (MDS-UPDRS-self), Parkinson Anxiety Scale (PAS), Ruminative Response Scale (RRS), List of external stressors	Higher levels of stress and anxiety.	−
Chagué et al. (2020) [57]	Congestive heart failure	Self-reported well-being Psychological distress Heart failure symptoms Health care access	Psychological distress --> Kessler 6 score (K6)	21.8% reported a decrease in well-being. 18.5% reported psychological distress. 21.8% reported an increase in health failure symptoms. Significant reduction in health care access.	−
Elran-Barak et al. (2020) [36]	Chronic diseases	(Change in) physical self-reported health (SRH) (Change in) mental physical self-reported health (SRH) Loneliness	Adapted Medical Outcomes Study-Short Form 36 items (SF-36 MOS)	47.2% reported decline in physical SRH. 14.6% reported a bad/very bad current physical SRH. 50.5% reported a decline in mental health. 14.2% reported a bad/very bad current mental health. Significant decline in level of loneliness.	−
Saqib et al. (2020) [44]	Chronic diseases	Self-reported overall health	2-Likert scale question	44.75% reported an effect on self-reported overall health.	−
Umucu et al. (2020) [48]	Self-reported disabilities and chronic conditions	Perceived stress Coping Well-being Depression and anxiety	Perceived stress questionnaire-8, Brief COPE, PERMA-Profiler, Patient Health Questionnaire-4	Small negative impact on well-being: moderate level of stress, depression and anxiety during the COVID pandemic.	−
Havermans et al. (2020) [38]	Cystic Fibrosis	Emotional well-being Changes in behavior or worries about CF	2-point Likert scale	Patients reported more sadness, discouragement, feelings of helplessness, perception of deterioration and difficulty with adhering to their routine.	−
Chiaravalloti et al. (2020) [58]	Progressive Multiple Sclerosis	Change in level of depression, anxiety, overall quality of life	COVID Impact survey HADS Beck Depression Inventory-II (BDI-II) Multiple Sclerosis Impact Scale EuroQol	Increased anxiety and depression. No difference in MS symptomatology. No significant difference on BDI-II. Significant increase in HADS-depression score, but no differences in HADS- anxiety scale or EQ5D scales.	−
Endstrasser et al. (2020) [56]	Osteoarthritis	Change in pain and mental health	Visual analogue scale (VAS), Western Ontario and McMaster Universities Osteoarthritis Index (WOMAC), Short-Form Health Survey (SF-12)	VAS and WOMAC scores increased significantly during lockdown. The mental health component remained largely unchanged.	−
Van de Venis et al. (2020) [49]	Hereditary spastic paraplegia	Change in psychological stress	5-Likert scale question	43% reported an increase in psychological stress.	−
Guo et al. (2020) [37]	Skin diseases	Perceived stress Anxiety Depression Quality of life	VAS, Perceived Stress Scale 14 item (PSS-14) Generalized Anxiety Disorder 7 item (GAD-7), Patient Health Questionnaire 9 item (PHQ-9), Dermatology Life Quality Index (DLQI)	Increased symptoms of anxiety and depression. Significant impaired mental well-being and quality of life.	−

Note: * Change in one or more constructs related to well-being. A negative change (−) indicates a decrease or decline in one or more well-being constructs during the COVID-19 pandemic compared with before the start of the pandemic. Well-being = WB.

**Table 6:** Different well-being constructs used in the included studies.

Author and Year	Anxiety	Depression	Loneliness	Mental Health	Overall Health	Pain	Quality of Life	Stress	Well-Being	Change in Well-Being
Sankar et al. (2020) [43]	✓							✓		−
Schirinzi et al. (2020) [45]		✓								−
Shalash et al. (2020) [46]				✓						−
Van der Heide et al. (2020) [50]								✓		−
Chagué et al. (2020) [57]								✓	✓	−
Elran-Barak et al. (2020) [36]			✓	✓						−
Saqib et al. (2020) [44]					✓					−
Umucu et al. (2020) [48]	✓	✓						✓	✓	−
Havermans et al. (2020) [38]		✓						✓	✓	−
Chiaravalloti et al. (2020) [58]	✓	✓								−
Endstrasser et al. (2020) [56]				✓		✓				−
Van de Venis et al. (2020) [49]								✓		−
Guo et al. (2020) [37]	✓	✓					✓	✓		−

Note: Change in one or more constructs related to well-being. A negative change (−) indicates a decrease or decline in one or more well-being constructs during the COVID-19 pandemic compared with before the start of the pandemic.

## Data Availability

No new data were created or analyzed in this study. Data sharing is not applicable to this article.

## Supplementary Materials

The following are available online at https://www.mdpi.com/article/10.3390/ijerph18126342/s1 Table S1: Physical activity pre and during lockdown.

## Appendix A

**Table A1:** Details of the search strategies.

Database	Search Strategy
**Pubmed**	( (COVID-19 [tiab] OR Sars-CoV-2 [tiab] OR coronavirus [tiab] OR corona virus [tiab]) AND (“Physical activity” [tiab] OR sport [tiab] OR sports [tiab] OR exercise [tiab] OR exercising [tiab] OR “physical training” [tiab] OR “physical performance” [tiab]) OR (COVID-19 [tiab] OR Sars-CoV-2 [tiab] OR coronavirus [tiab] OR corona virus [tiab]) AND (Sedentary behavior [tiab] OR sitting activity [tiab] OR Sedentary inactivity [tiab]) OR (COVID-19 [tiab] OR Sars-CoV-2 [tiab] OR coronavirus [tiab] OR corona virus [tiab]) AND (Well-being [tiab] OR Wellness [tiab] OR Wealth [tiab] OR Welfare [tiab]) )
**CINAHL**	( (AB (COVID-19 OR Sars-CoV-2 OR coronavirus OR corona virus) OR TI (COVID-19 OR Sars-CoV-2 OR coronavirus OR corona virus)) AND ( (AB (Physical activity OR Sport OR sports OR Exercise OR exercising OR Physical training OR Physical performance) OR TI (Physical activity OR Sport OR sports OR Exercise OR exercising OR Physical training OR Physical performance)) OR (AB (Sedentary behavior OR Sitting activity OR Sedentary inactivity) OR TI (Sedentary behavior OR Sitting activity OR Sedentary inactivity)) OR (AB (Well-being OR Wellness OR Wealth OR Welfare) OR TI (Well-being OR Wellness OR Wealth OR Welfare)) ) )
**PsycInfo**	( (AB (COVID-19 OR Sars-CoV-2 OR coronavirus OR corona virus) OR TI (COVID-19 OR Sars-CoV-2 OR coronavirus OR corona virus)) AND ( (AB (Physical activity OR Sport OR sports OR Exercise OR exercising OR Physical training OR Physical performance) OR TI (Physical activity OR Sport OR sports OR Exercise OR exercising OR Physical training OR Physical performance)) OR (AB (Sedentary behavior OR Sitting activity OR Sedentary inactivity) OR TI (Sedentary behavior OR Sitting activity OR Sedentary inactivity)) OR (AB (Well-being OR Wellness OR Wealth OR Welfare) OR TI (Well-being OR Wellness OR Wealth OR Welfare)) ) )
**Embase**	( (‘covid 19’:ab,ti OR ‘sars cov 2’:ab,ti OR coronavirus:ab,ti OR ‘corona virus’:ab,ti) AND (‘physical activity’:ab,ti OR sport:ab,ti OR sports:ab,ti OR exercise:ab,ti OR exercising:ab,ti OR ‘physical training’:ab,ti OR ‘physical performance’:ab,ti) OR (‘covid 19’:ab,ti OR ‘sars cov 2’:ab,ti OR coronavirus:ab,ti OR ‘corona virus’:ab,ti) AND (‘sedentary behavior’:ab,ti OR ‘sitting activity’:ab,ti OR ‘sedentary inactivity’:ab,ti) OR (‘covid 19’:ab,ti OR ‘sars cov 2’:ab,ti OR coronavirus:ab,ti) AND (‘well being’:ab,ti OR wellness:ab,ti OR wealth:ab,ti OR welfare:ab,ti) AND english:la AND [2019–2020]/py )

**Table A2:** List of excluded articles during full-text screening.

Reference	Exclusion Criteria
Balducci and Coccia (2020) [65]	Study is a commentary (out of study design).
Bonora et al. (2020) [66]	Study reported different outcomes (out of outcomes).
Boyle et al. (2020) [67]	Study is a commentary (out of study design).
Chung et al. (2020) [68]	Study has a too young population (out of population).
Cuschieri and Grech (2020) [69]	Study is a literature study (out of study design).
Fernandez-del-Valle et al. (2020) [70]	Study is a commentary (out of study design).
Giebel et al. (2020) [71]	Study reported effects in dementia (out of population).
Hall and Church (2020) [72]	Study is a review (out of study design).
Hudson and Sprow (2020) [73]	Study is a commentary (out of study design).
Jakiela et al. (2020) [74]	Study is a recommendation (out of study design).
Leung et al. (2020) [75]	Study is a review (out of study design).
López-Sánchez et al. (2020) [76]	Study is published on 10 October (out of publish date).
Mobasheri (2020) [77]	Study is an editorial (out of study design).
Moghadasi (2020) [78]	Study did not make a comparison with situation before the COVID-19 pandemic (out of comparison).
Motl et al. (2020) [79]	Study is an editorial (out of study design).
Orhurhu et al. (2020) [80]	Study is an editorial (out of study design).
Palmer et al. (2020) [81]	Study is a review (out of study design).
Peçanha et al. (2020) [82]	Study is a review (out of study design).
Quinn et al. (2020) [83]	Study is an implementation study (out of study design).
Rhodes et al. (2020) [84]	Study is a recommendation (out of study design).
Sennott et al. (2020) [85]	Study is a commentary (out of study design).
Speretta and Leite (2020) [86]	Study is an editorial (out of study design).
Tornese et al. (2020) [87]	Study has a too young population (out of population).
Verma et al. (2020) [88]	Study has a too young population (out of population).
