# Supplementary material for: Physical Activity, Sedentary Behavior and Well-Being of Adults with Physical Disabilities and/or Chronic Diseases during the First Wave of the COVID-19 Pandemic: A Rapid Review
Source: Int J Environ Res Public Health. 2021 Jun 11;18(12):6342. doi: 10.3390/ijerph18126342 (PMC8296179; doi:10.3390/ijerph18126342)
Supplement: Supplementary file 1 [file ijerph-18-06342-s001.zip › ijerph-1234061-supplementary.pdf]

International Journal of
Environmental Research and Public Health

MDPI

Table S1. Physical activity pre and during lockdown.

Author and Year	PA type and units of measurement	PA Pre-Lockdown Mean (SD)	PA During Lock-down Mean (SD)	Change in PA	P value (if applica-ble)	Lockdown restrictions
Barone et al. (2020) [27]	*Change in PA - % participants*<br>High increase<br>Low increase<br>No change<br>Low reduction<br>High reduction	N.A.	2.29%<br>4.64%<br>33.57%<br>14.70%<br>44.8%	N.A.	N.A.	"Decentralized (defined by states and municipalities) and delayed, the measures to contain the SARS-CoV-2 spread in Brazil, and reflected on mobility reduction, was experienced by 95.1% of this study's respondents in different degrees (with 26.9% never going outside their homes)"
Khader et al. (2020) [34]	*Change in PA - % participants*<br>Increased<br>No change<br>Decreased	N.A.	69%<br>25%<br>6%	N.A.	N.A.	"A series of lockdowns starting from March 24, 2020 were imposed on people in the country to break the chain of virus transmission [4]. Further, United Nations and WHO have praised India's response to the pandemic as 'comprehensive' and 'robust'. However, since the start of "unlocking" and easing of restrictions from June 1st, India is witnessing an exponential rise in COVID-19 cases indicating the possibility of 'community transmission'.
Yan et al. (2020) [46]	*Change in PA - % total participants*<br>Increased a lot<br>Increased a little<br>No change<br>Decreased a little<br>Decreased a lot<br><br>*Change in PA - % participants without diabetes*<br>Increased a lot<br>Increased a little<br>No change<br>Decreased a little<br>Decreased a lot<br><br>*Change in PA - % participants with diabetes*<br>Increased a lot	N.A.	17.8%<br>25.0%<br>24.6%<br>17.3%<br>15.2%<br><br>16.0%<br>25.2%<br>25.0%<br>17.9%<br>16.0%<br><br>44.6%<br>23.1%<br>19.5%<br>8.2%	N.A.		"China implemented a lockdown of Wuhan in late January 2020 to contain the spread of COVID-19. China is slowly beginning to reopen since lifting the lockdown in mid-May."

Increased a little	4.6%	
No change		
Decreased a little		<0.001
Decreased a lot	1.2 (1.5)	
	1.1 (1.4)	
PA - hours per day	2.0 (2.0)	
Total participants		
Participants without diabetes		<0.001
Participants with di-abetes	59.2%	
	58.1%	
	73.8%	
PA 150 minutes per week - % participants		
Total participants		
Participants without	42.9%	
diabetes	9.3%	
Participants with di-	20.5%	
abetes	14.4%	
	12.9%	
Frequency of exercise - % participants with-out diabetes		
Never/Rarely	46.5%	
<1 time per week	8.5%	
1–2 time per week	26.5%	
3–5 time per	10.3%	
Daily	8.2%	
Frequency of exercise - % participants with		
diabetes	40.0%	
Never/Rarely	23.9%	
<1 time per week	11.8%	
1–2 time per week	31.5%	
3–5 time per	40.6%	
Daily		
Type of exercise - %		
participants without	43.4%	
diabetes	47.7%	
Walking / slow	38.8%	
walking	23.4%	

	Quick walking / square dance		25.6%			
	Tai Chi/ Qigong / Ba Duan Jin					
	Other indoor exercises					
	No exercise					
	Type of exercise - % participants with diabetes					
	Walking / slow walking					
	Quick walking / square dance					
	Tai Chi/ Qigong / Ba Duan Jin					
	Other indoor exercises					
	No exercise					
Assaloni et al. (2020) [47]	*Minutes of exercise*	66 (42)	38 (31)	−28		“In accordance to Istituto Superiore della Sanità (ISS), the Italian Government implemented extraordinary measures to limit viral transmission throughout people and the territory, imposed national quarantine, reduced social interaction and travelling and ‘‘stay at home” as a basic means of limiting people’s exposure to the virus.
	Number of steps	12606 (5026)	4760 (3145)	−7846		
	Type of exercise - % particitpants				<0.001	
	Individual sport	35.7%	-			
	Team sport	4.5%	-		<0.001	
	Fitness	10.4%	-			
	Resistance training	3.9%	-			
	In autonomy	36.4%	82.5%	+46.1%		
	None	9.1%	17.5%	+8.4%		
khare et al. (2020) [48]	*Change in type - % participants*	N.A.		N.A.	N.A.	“Temporary lock down of country along with all precautions advised like social distancing and social isolation.”
	Total		80.42%			
	Male		81.32%			
	Female		78.85%			
	Change in timing - % participants					
	Total		72.72%			
	Male		73.63%			
	Female		71.15%			

	Change in duration - % participants<br>Total<br>Male<br>Female		60.84%<br>56.04%<br>69.23%			
Munekawa et al. (2020) [35]	*Change in exercise - Likert scale (0: considerably reduced, 5: no change, 10: considerably increased)*<br><br>*Decreased exercise - % participants*	N.A.	3.7 (2.0)<br><br>53.96%	N.A.	N.A.	"In Japan, a state of emergency, with request-based measures of encouraging the populace to remain at home and businesses to limit operations, was declared on April 7, 2020. Therefore, the Japanese are also becoming more restrictive in their behavior."
Ruiz-Roso et al. (2020) [37]	*Moderate PA – minutes per week*<br>Male<br>Female<br>44–63 years old<br>64–77 years old<br>BMI: 25.0–<30 kg/m²<br>BMI: 30–<35 kg/m²<br>BMI: 35–<40 kg/m²<br>Capillary HbA1c: <6,5%<br>Capillary HbA1c: ≥6,5%<br><br>*Walking – minutes per week*<br>Male<br>Female<br>44–63 years old<br>64–77 years old<br>BMI: 25.0–<30 kg/m²<br>BMI: 30–<35 kg/m²<br>BMI: 35–<40 kg/m²<br>Capillary HbA1c: <6,5%<br>Capillary HbA1c: ≥6,5%	60.5<br>44.5<br>190.6<br>68.2<br>81.3<br>102.9<br>124.0<br>125.7<br>57.2<br><br>363.2<br>268.4<br>231.9<br>339.9<br>434.7<br>270.4<br>214.8<br>291.3<br>289.5<br><br>5.2<br>5.6<br>5.9<br>5.2<br>6.0<br>5.2	20.6<br>21.2<br>33.8<br>48.4<br>32.7<br>60.9<br>26.3<br>43.6<br>40.3<br><br>108.8<br>128.3<br>89.7<br>124.8<br>138.0<br>113.3<br>95.9<br>127.2<br>85.4<br><br>7.7<br>6.8<br>7.3<br>7.3<br>7.5<br>6.7	−39.9<br>−23.3<br>−156.8<br>−19.8<br>−48.6<br>−42.0<br>−97.7<br>−82.1<br>−16.9<br><br>−254.4<br>−140.1<br>−142.2<br>−215.1<br>−296.7<br>−157.1<br>−118.9<br>−164.1<br>−204.1<br><br>+2.5<br>+1.2<br>+1.4<br>+2.1<br>+1.5<br>+1.5	0.0007<br>0.0005<br>0.0119<br>0.3694<br>0.2803<br>0.0658<br>0.0933<br>0.0118<br>0.5591<br><br>0.0006<br>0.0005<br>0.0007<br><0.0001<br>0.0037<br>0.0002<br>0.0142<br>0.0003<br>0.0004<br><br><0.0001<br>0.0213<br>0.0332<br><0.0001<br>0.0516<br>0.001	"The Spanish government released on the 14th of March an executive order to implement a state of alarm, when strict lockdown measures such as social distancing and complete confinement at home were first imposed."

	Sitting – hours per week<br>Male<br>Female<br>44–63 years old<br>64–77 years old<br>BMI: 25.0–<30 kg/m²<br>BMI: 30–<35 kg/m²<br>BMI: 35–<40 kg/m²<br>Capillary HbA1c: <6,5%<br>Capillary HbA1c: ≥6,5%	5.4<br>5.4<br>5.0	7.2<br>6.9<br>7.2	+1.8<br>+1.5<br>+2.2	0.0043<br><0.0001<br>0.0002	
Sankar et al. (2020) [38]	*Change in PA - % participants*<br>Increased<br>Same as before<br>Decreased	N.A.	2.7%<br>82.7%<br>14.5%	N.A.	N.A.	"In Pathanamthitta district of South Kerala, the lockdown was enforced on March 16, 2020, one week earlier than the nationwide 21-day lockdown 1.0 which was announced on March 24, 2020. This was followed by lockdown 2.0 and 3.0 which ended on May 17, 2020."
Brown et al. (2020) [28]	*Change in exercise - % participants*<br>Conducted via alternative method(s)<br>Postponed<br>Cancelled<br>Not applicable	N.A.	41%<br>7,9%<br>21%<br>30%	N.A.	N.A.	N.A.
Schirinzi et al. (2020) [40]	*Playing sports - % participants*	80%	81%	+1%	N.A.	N.A.
Shalash et al. (2020) [41]	*PA decline - % participants*<br>*PA decline*	N.A.	68.4%	N.A.	0.002	N.A.
Song et al. (2020) [42]	*PA frequency – times per week*<br>*PA duration – hours per session*<br>*PASE leisure part score*<br>*Patterns of exercise - % participants*<br>None	5.0<br>1.0<br>26.4<br>7%<br>58%<br>7%<br>23%<br>5%	5.0<br>0.7<br>12.8<br>22%<br>60%<br>12%<br>5%<br>1%	0,0<br>−0.3<br>−13,6<br>+15%<br>+2%<br>+5%<br>−18%<br>−4%	0.011<br>0.003<br><0.001<br><0.001<br>1.000<br>0.315<br><0.001<br>0.625	"In Korea, the first COVID-19 was reported on January 21, 2020 and the number of daily new cases increased to 909 on February 29, 2020. The Korean government placed enhanced social distancing (staying at home and refraining from going out as much as possible) from March 22, 2020 to May 19th, 2020, and then changed to routine social distancing."

	Outdoor-solo<br>Indoor-solo<br>Exercise at sports facilities<br>Group exercise					
Van der Heide et al. (2020) [45]	*Change in PA - % participants*<br>More active<br>Equally active<br>Less active	N.A.	20.4%<br>33.0%<br>46.6%	N.A.	N.A.	"The first COVID-19 case in the Netherlands was confirmed on February 27, 2020. March 15–20.: Closure of hospitality, schools, nursing homes. No visitors in hospitals. March 23.: Request to stay at home. Only essential traveling. June 1.: Reopening of hospitality and all schools."
Chagué et al. (2020) [52]	*Decrease in PA - % participants*<br>Total<br>Men<br>Women	N.A.	41.9%<br>33.3%<br>55.1%	N.A.	N.A.	"Limitations in access to care"
Vetrovsky et al. (2020) [54]	*Average daily step count - 3 weeks before lockdown vs. 3 weeks within lockdown*	N.A.	N.A.	−1134 (189)<br>= −16.2%	<0.001	"In the Czech Republic, the first case of COVID-19 was reported on 1 March 2020, and since 16 March, the Czech government approved a nationwide quarantine that prohibited movement in public spaces except under special circumstances, which included travelling to and from work and necessary journeys to procure food and supplies; notably, going outside for a walk in a park or the countryside was allowed."
Malanchini et al. (2020) [49]	*Device-derived daily patient activity level - hour per day*<br>Reference period<br>Pre lockdown period<br>Lockdown period	2.18 (1.3)<br>2.14 (1.3)	1.62 (1.2)	−26.1% (25.0%)	<0.0001	N.A.
Sassone et al. (2020) [50]	*Time spent in movement based on accelerometry - hours per day*	1.6 (0.5)	1.2 (0.3)	−25%	0.0001	N.A.
	Time spent in movement based on accelerometry without very inactive patients - hours per day	2.0 (0.6)	1.5 (0.4)	−25%	0.0001	
Cransac-Miet et al. (2020) [29]	*Change in PA - % total participants*<br>>25% increase<br>No change<br>>25% decrease	N.A.	8.2%<br>46.7%<br>45.1%	N.A.	N.A.	N.A.

	Change in PA - % participants in urban area<br>>25% increase<br>No change<br>>25% decrease<br><br>*Change in PA - % participants in rural area*<br>>25% increase<br>No change<br>>25% decrease		8.8%<br>39.4%<br>51.8%<br><br>7.6%<br>57.0%<br>35.4%			
Elran-Barak et al. (2020) [31]	*Frequency of PA (>0.5 hour) - times per week*	3.5 (2.4)	2.8 (2.4)	−0.7	<0.001	"In an effort to contain the COVID-19 outbreak, the Israeli government announced a number of new restrictions aimed at reinforcing social distancing. On March 12, Israel announced that all universities and schools would close. On March 16, all non-critical government and local authority workers were placed on paid leave, and private sector firms were required to reduce the staff presence in the workplace. On March 19, Prime Minister Benjamin Netanyahu declared a national state of emergency, saying that existing restrictions would henceforth be legally enforceable, and violators would be fined. Israelis were not allowed to leave their homes unless absolutely necessary, putting a near-lockdown into effect. Essential services - including grocery stores, pharmacies, and banks – remained open, but people were prohibited from venturing more than 100 m from their homes, apart from under certain circumstances (e.g., stocking up on food and medicine). Non-essential stores were required to close, and parks were to remain shut. People were required not to participate in any social gatherings and to limit face-to-face interactions with individuals outside the immediate household."
Saqib et al. (2020) [39]	*Continue daily exercise - % partcipants didn't continue*	N.A.	66%	N.A.	N.A.	N.A.
Havermans et al. (2020) [33]	*Increase in exercise - % participants*<br>Patients with CF<br>CF with LTX<br>Parents of children with CF	N.A.	47.4%<br>46%<br>23.3%	N.A.	N.A.	"A national lockdown was enforced by the Belgian government on March 18, 2020. People had to stay indoors as much as possible which meant they had to work from home. Schools and public places were closed, only food stores and pharmacies were opened to a limited number of people at the same time and non-essential movements, except for walking, running and cycling, were forbidden."
Radtke et al. (2020) [36]	*Change in PA - % total participants*<br>More frequently<br>No change<br>Less frequently	N.A.	23.0%<br>32.2%<br>44.8%	N.A.	N.A.	"The COVID-19 pandemic developed with regional differences."

	Change in PA - %		25.4%			
	non-LTX participants		45.1%			
	More frequently		45.1%			
	No change					
	Less frequently					
Endstrasser et al. (2020) [51]	*SF-12 Physical Component Summary score*					N.A.
	Pre lockdown					
	During lockdown	37.89 (8.92)	37.36 (9.08)	−0.53	0.204	
	Post lockdown		35.48 (9.62)	−2.41 & −1.88	0.026 & 0.071	
Di Stefano et al. (2020) [30]	*MET total PA - minutes per week*					N.A.
	Patients with neuromuscular disease	901.3 (1299.6)	400.6 (1088.5)	−500.7 (705.7)	<0.0001	
	Healthy controls	4506.5 (7600.1)	2362.3 (4498.9)	−2144.3 (8630.7)	<0.0001	
	MET vigorous intensity PA - minutes per week					
	Patients with neuromuscular disease	70.1 (361.9)	37.1 (303.9)	−33 (219.2)	0.69	
	Healthy controls	2081.8 (4945.3)	861.9 (1662.9)	−1219.9 (4920.8)	0.04	
	MET moderate intensity PA - minutes per week					
	Patients with neuromuscular disease	263.2 (606.9)	146.9 (450.6)	−116.2 (323.2)	0.07	
	Healthy controls	1153.3 (2424.6)	925.4 (3675.6)	−227.87 (4076.9)	0.01	
	MET walking intensity PA - minutes per week					
	Patients with neuromuscular disease	547.7 (733.2)	211.9 (534)	−149.3 (426.8)	<0.0001	
	Healthy controls	1271.5 (2703.6)	574.9 (1731.3)	−1447.8 (7611.3)	<0.0001	
	Moderate-to-vigorous PA - minutes per week					
	Patients with neuromuscular disease	333.3 (483.8)	184 (440.3)	−149.3 (426.8)	0.04	
	Healthy controls	3235.7 (3684.7)	1787.3 (2669.3)	−1447.8 (7611.3)	0.001	

Van de Venis et al. (2020) [44]	*Change in PA - % participants*<br>Strong increase<br>Mild increase<br>No change<br>Mild decrease<br>Strong increase	N.A.	0%<br>7%<br>19%<br>41%<br>33%	N.A.	N.A.	"Partial lockdown in the Netherlands"
Al-Hashel et al. (2020) [26]	*Lack of regular exercise - % participants*	N.A.	79.7%	N.A.	N.A.	N.A.

Note: physical activity, PA.
